# Magnetic resonance imaging diagnosis of non-mass enhancement of the breast

**DOI:** 10.1007/s10396-023-01290-2

**Published:** 2023-02-18

**Authors:** Kazunori Kubota, Mio Mori, Tomoyuki Fujioka, Kaoru Watanabe, Yuko Ito

**Affiliations:** 1 Department of Radiology, Dokkyo Medical University Saitama Medical Center, 2-1-50 Minamikoshigaya, Koshigaya, Saitama 343-8555 Japan; 2 Department of Diagnostic Radiology, Tokyo Medical and Dental University, Tokyo, Japan

**Keywords:** Breast cancer, MRI, BI-RADS, Non-mass enhancement

## Abstract

Breast Imaging Reporting and Data System magnetic resonance imaging (BI-RADS-MRI) classifies lesions as mass, non-mass enhancement (NME), or focus. BI-RADS ultrasound does not currently have the concept of non-mass. Additionally, knowing the concept of NME in MRI is significant. Thus, this study aimed to provide a narrative review of NME diagnosis in breast MRI. Lexicons are defined with distribution (focal, linear, segmental, regional, multiple regions, and diffuse) and internal enhancement patterns (homogenous, heterogeneous, clumped, and clustered ring) in the case of NME. Among these, linear, segmental, clumped, clustered ring, and heterogeneous are the terms that suggest malignancy. Hence, a hand search was conducted for reports of malignancy frequencies. The malignancy frequency in NME is widely distributed, ranging from 25 to 83.6%, and the frequency of each finding varies. Latest techniques, such as diffusion-weighted imaging and ultrafast dynamic MRI, are attempted to differentiate NME. Additionally, attempts are made in the preoperative setting to determine the concordance of lesion spread based on findings and the presence of invasion.

## Introduction

Breast magnetic resonance imaging (MRI) has the highest diagnostic accuracy in breast imaging and is used for screening, preoperative examination, breast lesion strategy determination, and chemotherapy response evaluation [1]. In recent years, diagnostic methods based on Breast Imaging Reporting and Data System (BI-RADS), which is published by the American College of Radiology, have been used [2]. Lesions are classified as mass, non-mass enhancement (NME), or focus. NME is an area of enhancement that is not characterized as a mass, which is a lesion occupying space in three dimensions, and is different from focus, which is a point-like nonspecific enhancement. Understanding the diagnostic terminology of other modalities is important for the future of ultrasound diagnosis, although BI-RADS ultrasound does not currently have the concept of non-mass. This study provides a narrative review of NME diagnosis in breast MRI.

### Introduction of BI-RADS-MRI

Imaging and reading methods for breast MRI were not standardized before the introduction of BI-RADS. The first edition of BI-RADS-MRI was published in 2003, initiating the diagnosis using the common lexicon [3]. Evidence and expert opinion were compiled, and the second edition of BI-RADS-MRI was revised in 2013 [4]. The term “non-mass-like enhancement” was changed to “non-mass enhancement,” and other terminologies were revised in the second edition. Therefore, when reading the literature, consider that the diagnosis based on previous imaging and reading methods is different from the current diagnosis.

BI-RADS describes lesions using a unified terminology of findings, which are called lexicons, and assessment categories are determined based on the combination of lexicons (Table 1).

**Table 1:** BI-RADS categories and recommended management

Category 0—needs additional imaging evaluation (use of MRI not recommended)
Category 1—negative
Category 2—benign
Category 3—probably benign (< 2% chance of malignancy), short interval (usually 6 months) follow-up suggested
Category 4—suspicious (2–95% chance of malignancy), biopsy should be considered
4A—low suspicion for malignancy (2–10% chance of malignancy)
4B—moderate suspicion for malignancy (10–50% chance of malignancy)
4C—high suspicion for malignancy (50–95% chance of malignancy)
Category 5—highly suggestive of malignancy (> 95% chance of malignancy), biopsy should be considered
Category 6—known biopsy-proven malignancy

Categorization is based on assessment, but it is closely related to management. Follow-up at 6 months is recommended for category 3, while pathological diagnosis by needle biopsy is for categories 4 and 5.

**Fig. 1:**
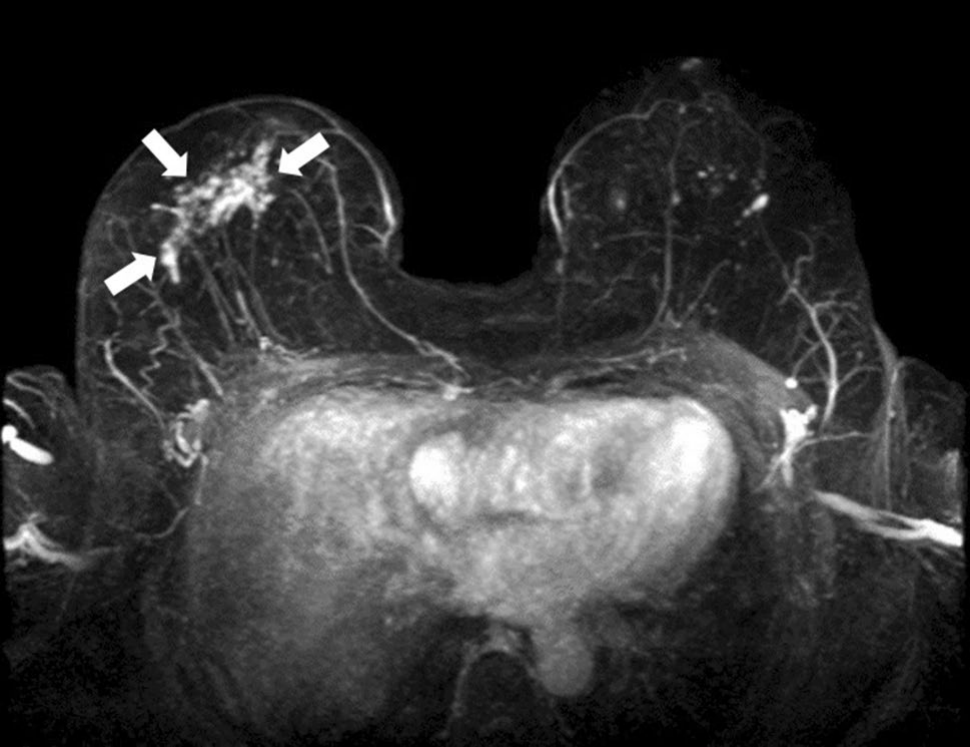
A female patient in her 70 s is an example of a case of non-mass enhancement with a clumped pattern and segmental distribution. The pathological diagnosis was right breast ductal carcinoma in situ

**Fig. 2:**
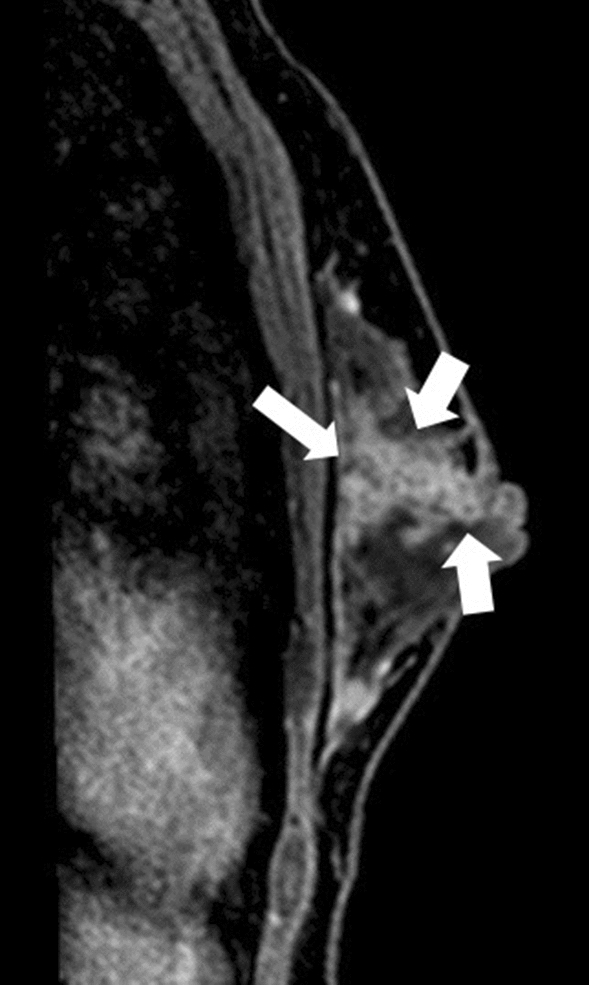
A female patient in her 30 s is an example of a case of non-mass enhancement with clustered ring enhancement and segmental distribution. The pathological diagnosis was right breast ductal carcinoma in situ, extending to the nipple

**Fig. 3:**
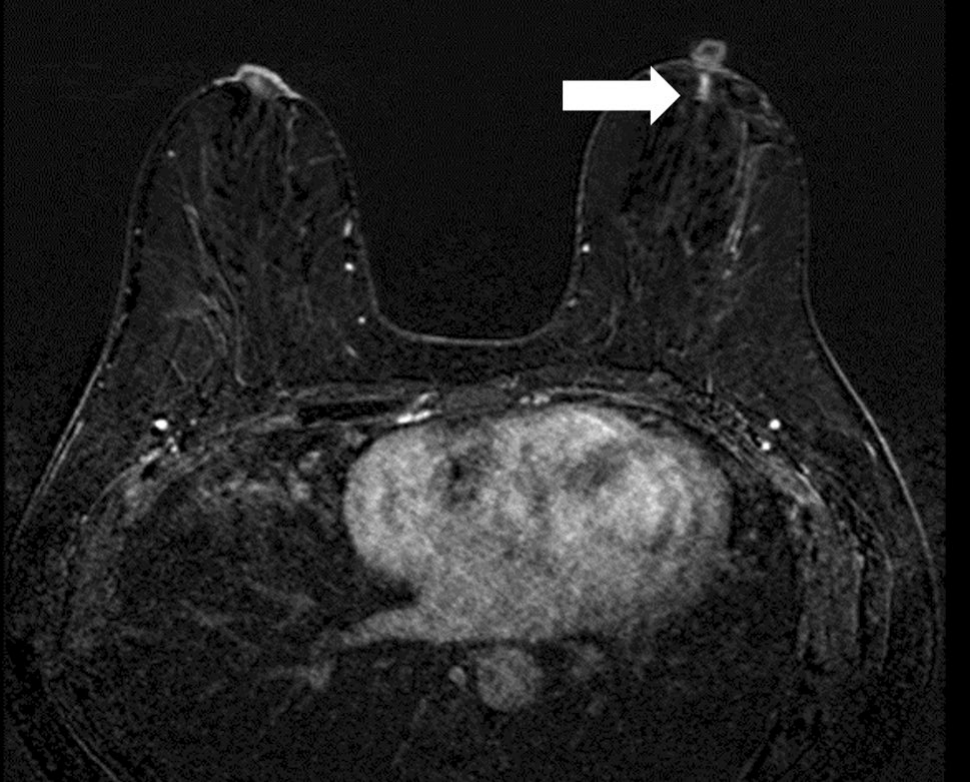
A female patient in her 40 s is an example of a case of non-mass enhancement with a homogenous and linear distribution. Needle biopsy showed a pathological diagnosis of left breast adenoma

Lexicons are defined with distribution (focal, linear, segmental, regional, multiple regions, and diffuse) and internal enhancement patterns (homogenous, heterogeneous, clumped, and clustered ring) for NME. Among these, linear, segmental, clumped, clustered ring, and heterogeneous are the terms that suggest malignancy. In particular, these NMEs are intended for ductal carcinoma in situ (DCIS). Figures 1, 2, 3 show a typical case from our experience.

Conversely, confirming the diagnosis based on images alone is quite difficult. Benign lesions may overlap, such as mastopathy, intraductal papilloma, atypical ductal hyperplasia, flat epithelial atypia, and sclerosing adenopathy. Therefore, proper lesion categorization and needle biopsy diagnosis, including vacuum-assisted biopsy and core needle biopsy, is necessary for lesions in categories 4 and 5. The pathology results should be confirmed to be consistent with the imaging findings if they are benign when a needle biopsy is performed on a lesion suspected of malignancy. NME can be associated with invasive ductal carcinoma (IDC) or invasive lobular carcinoma, but determining the exact presence of mixed invasiveness in NME is difficult.

### Background parenchymal enhancement (BPE) and NME

Previously, any enhancement effect was suggestive of some kind of lesion because of the high diagnostic capability of breast MRI. However, normal mammary gland tissue can now show enhancement due to hormonal effects, which are treated as BPE. BPE is evaluated in the early contrast phase and classified into four levels: minimal, mild, moderate, and marked, and with symmetry or asymmetry. The examination should be performed upon screening because BPE is considered as most vulnerable 7–14 days after the onset of menstruation due to the influence of the hormonal cycle. Therefore, the test should be performed upon screening and other purposes. Conversely, treatment should not be delayed by adjusting the examination schedule if the MRI is performed as a preoperative examination.

When BPE is asymmetric, it may be confused with NME. When BPE is more focal, regional, or asymmetric, it has been associated with a higher likelihood of a BI-RADS 3 assessment (> 2% likelihood of malignancy). Alternatively, false positives are common when asymmetric BPE is biopsied. In nonsurgical (i.e., screening) cases, 6-month follow-up MR imaging as BI-RADS category 3 is a substitute for biopsy when BPE is favored over a pathologic process [5].

Chikarmane et al. reported that 77 (20%) of 386 breast MRIs previously read as NME should be discriminated as BPE when read according to BI-RADS 2013 (one of them had breast cancer) [6].

### Determination of benign and malignant lesions

BI-RADS recommends a biopsy for a category 4 lesion with a ≥ 2% likelihood of malignancy. Therefore, the lesion is considered category 4 or higher if any of the findings suggest malignancy. However, many NMEs are benign lesions, noninvasive, and not immediately life-threatening. Detection and treatment of non-life-threatening lesions are called overdiagnosis, and some malignant lesions found in NME may well be overdiagnosis. Therefore, we should not simply biopsy category 4 lesions, but also consider the possibility of malignancy and the degree of invasive cancer, and inform the patient of the possibility of a follow-up.

Lesions that are difficult to determine and must be followed up with NME in BI-RADS are classified as category 3, although they have no evidence.

### Findings and malignant potential

Shao et al. published a meta-analysis of non-mass-like lesions in 2013 [7]. They reported a sensitivity of 50% and specificity of 80% for non-mass-like enhancement in 14 studies and 858 cases. The positive predictive value (PPV) was 67%, especially in segmental and clustered rings. However, the studies included in this report are somewhat old (2002–2011) and pre-date the publication of BI-RADS-MRI and its second edition because the term “non-mass-like lesion” was used in the previous BI-RADS edition. The results cannot be applied to current diagnoses because they include imaging methods that are no longer standard, such as unilateral and supine scanning, as well as those from a time when the diagnostic criteria were ambiguous.

Therefore, we conducted a hand search to examine NME reports based on the BI-RADS-MRI 2013 edition in recent studies [8–12]. Table 2 shows the PPV and number of cases (malignant/number of findings) by lexicons. The malignancy frequency of NME is widely distributed, ranging from 25 to 83.6% because of the different settings of the control groups and the extent to which they are included in BPE. Internal enhancement was highly variable, and even clustered ring and clumped lesions included a relatively large number of benign lesions. Segmental lesions are very likely to be malignant, while linear lesions are less likely to be malignant. Focal lesions seem to be less isointense but have a certain malignancy frequency. Showing a certain trend was impossible for regional, multiple regional, and diffuse, where the number of cases is small.

**Table 2:** The PPV and number of cases (malignant/number of findings) by lexicons

Author year	All NME	Homogenous	Heterogenous	Clustered ring	Clumped	Focal	Linear	Segmental	Regional	Multiple regional	Diffuse
Almeida 2016 [8]	44.6% (29/65)	12.5% (1/8)	5.6% (3/54)	N.A	100% (3/3)	28% (7/25)	36.4% (4/11)	80% (12/15)	25% (1/4)	100% (1/1)	N.A
Asada 2018 [9]	83.6% (178/213)	22.2% (2/9)	70% (21/30)	80% (20/25)	90.6% (135/149)	69.4% (25/36)	43.8% (7/16)	90.8% (138/152)	50% (1/2)	100% (4/4)	100% (3/3)
Aydin 2019 [10]	25% (33/132)	0% (0/14)	20.8% (5/24)	41.9% (13/31)	20% (12/60)	17.5% (7/40)	5.4% (2/37)	70.6% (12/17)	23.8% (5/21)	10% (1/10)	75% (3/4)
Lunkiewicz 2020 [11]	28.4% (19/67)	20% (2/10)	17.6% (3/17)	53.8% (7/13)	25.9% (7/27)	17.9% (5/28)	27.8% (5/18)	62.5% (5/8)	18.2% (2/11)	50% (1/2)	N.A
Liu 2022 [12]	47.5% (56/118)	0% (0/1)	31.9% (15/47)	86.4% (19/22)	45.8% (22/48)	40% (10/25)	7.7% (1/13)	64.9% (24/37)	37.5% (6/16)	55.6% (5/9)	55.6% (10/18)

Khoury et al. examined interobserver variability in the description and assignment of BI-RADS categories and revealed worse agreement in NME than in masses, although with some acceptable agreement [13]. Additionally, the frequency of benign and malignant concerning internal enhancement widely varies, possibly due to somewhat ambiguous definitions regarding the distinction between heterogeneous and homogenous.

Chen et al. examined 120 cases of linear enhancement and reported a PPV of 20.8%, with no significant difference due to internal enhancement or size difference [14]. The 2003 edition of BI-RADS-MRI used the term “ductal” to refer to intraductal lesions, but the 2013 edition no longer uses this term. Linear and ductal may still be confused, so we need to be careful.

Asada et al. revealed a PPV of 93.8% for the combination of clustered ring and segmental regarding diagnosis based on a combination of findings [9], while Aydin et al. revealed a PPV of 61.5%, with some discrepancy between the studies [10].

The division of BI-RADS category 4 into subcategories 4A, 4B, and 4C is not recommended in BI-RADS-MRI, but is allowed at individual institutions by consensus, and may be useful in determining treatment policies at each institution. Honda et al. classified category 4 lesions into three subcategories and analyzed the PPV of malignancy, with PPVs of subcategories 4A, 4B, and 4C of 1.8%, 11.8%, and 67.5%, respectively [15].

The Kaiser score is a well-known method of judgment based on a combination of terms, which presents the possibility of malignancy by adding points according to the findings [16]. However, it does not directly correspond to the lexicon of NME in BI-RADS. Such categorization has been shown to appropriately indicate the malignancy frequency in NMEs, which helps make medical decisions.

Some kinetics studies have been conducted, although BI-RADS does not require kinetics measurement at the NME [8, 10, 12]. NME diagnosis by kinetics is challenging because determining the location of the region of interest in small ductal or diffuse lesions is difficult and noninvasive cancers often have a persistent pattern. The results of the reports do not show a uniform trend.

### Other new diagnostic methods not used in BI-RADS are being investigated

Diffusion-weighted imaging (DWI) reflects the Brownian motion of water molecules, with a high signal in lesions with high cell proliferation and a low apparent diffusion coefficient (ADC). Therefore, DWI is considered useful in breast MRI for differentiating benign from malignant lesions and is often performed in combination with contrast-enhanced MRI. However, DWI has no fixed standards and has not yet been incorporated into BI-RADS. Regarding DWI, some have suggested that a low ADC value is indicative of malignancy [10, 12], while others have stated that NME discrimination remains difficult [17, 18]. Particularly, DWI has not yet been evaluated to a certain degree, but it may be useful in some cases.

Goto et al. studied ultrafast dynamic MRI and included 59 cases of NME, 37 cases of malignancy, and 22 cases of DCIS. Differences were found in two parameters that indicate the degree of early enhancement, time to enhance, and maximum slope between benign and malignant cases, which may be useful [19]. However, some benign cases were enhancing from a very early phase, and making a definite diagnosis remained difficult.

### Preoperative diagnosis

Size, presence of invasive cancer complications, and extension into the nipple need to be ascertained in the preoperative diagnosis.

Roque et al. measured the size of pure DCIS in a meta-analysis and revealed that most instances of DCIS were NME, and MRI accurately predicted the tumor size in 55% of cases. A meta-analysis revealed a 3.85-mm mean difference between MRI and pathology (95% confidence interval: -0.92–8.60), indicating that MRI is an accurate method for assessing pure DCIS size [20].

Maffuzu et al. reported a direct relationship between the incidence of microinvasion/invasion and tumor size, although a larger size was associated with more invasive cancer, and tumor sizes of DCIS were 2.5–3.5 cm in 10%, 3.6–4.5 cm in 57%, and 4.5–6 cm in 71% [21].

Machida et al. revealed three observer PPVs with clustered rings (PPVs: 54.5%, 54.5%, and 50.0%) and hypointense areas (PPVs: 63.6%, 61.5%, and 73.9%) as significantly associated with invasion. Clustered rings and hypointense areas that were integrated into heterogeneous structures showed significant associations with invasion (PPVs: 54.3%, 53.3%, and 51.8%) [22].

Oda et al. reported a 27% upgrade (DCIS to IDC) for non-mass, with a higher likelihood when palpable, although imaging lexicons, including clustered rings, did not show a consistent trend [23]. Further study is necessary to determine whether the imaging lexicons suggest invasion.

Bae SJ et al. reported that MRI adequately detected the presence or absence of cancer extension to the nipple. The PPV of papillary extension was 86% when NME involved the nipple, and only 7% had pathologic papillary extension when NME did not involve the nipple. The diagnostic accuracy rate was 88% [24].

## Conclusion

We have described NME, focusing on the BI-RADS-based diagnosis. BI-RADS uses lexicons for evaluation, but three important aspects should be understood. First, observers may have different perceptions regarding whether enhancement is a lesion or BPE. Second, the use of lexicons involves potential interobserver disagreement. Third, combining internal enhancement, distribution, and other findings in addition to a single term is important.

Providing further explanation on the use of lexicons is necessary to introduce appropriate training methods and reexamine the definition of terms as necessary. Therefore, continuous collection of results based on current BI-RADS terminology and expanding the evidence base are desirable. A large number of cases must be investigated and further validated to make a proper diagnosis. MRI is expected to become more useful in the future because of its high diagnostic performance. We hope that the accumulation of evidence and the utilization of new technologies will make MRI even more useful going forward. Therefore, breast MRI is expected to become an even more useful diagnostic method by collecting more evidence and utilizing new technologies.

## Declarations

### Informed conset

Informed consent was obtained from all patients whose cases were presented in this article.
